# Common *ALDH3A1* Gene Variant Associated with Keratoconus Risk in the Polish Population

**DOI:** 10.3390/jcm11010008

**Published:** 2021-12-21

**Authors:** Mariusz Berdyński, Piotr Krawczyk, Krzysztof Safranow, Beata Borzemska, Jacek P. Szaflik, Karolina Nowakowska-Żawrocka, Cezary Żekanowski, Joanna Giebułtowicz

**Affiliations:** 1 Laboratory of Neurogenetics, Department of Neurodegenerative Disorders, Mossakowski Medical Research Institute, Polish Academy of Sciences, 5 Pawińskiego Str., 02-106 Warsaw, Poland; m.berdynski@imdik.pan.pl (M.B.); beatab@imdik.pan.pl (B.B.); c.zekanowski@imdik.pan.pl (C.Ż.); 2 Department of Ophthalmology, Medical University of Warsaw, 13 Sierakowskiego Str., 03-709 Warsaw, Poland; piotrkrav@yahoo.fr (P.K.); jacek@szaflik.pl (J.P.S.); 3 Department of Biochemistry and Medical Chemistry, Pomeranian Medical University, 72 Powstańców Wlkp. Str., 70-111 Szczecin, Poland; chrissaf@mp.pl; 4 Department of Bioanalysis and Drugs Analysis, Faculty of Pharmacy, Medical University of Warsaw, Banacha 1 Str., 02-097 Warsaw, Poland; karolina.nowakowska2008@gmail.com

**Keywords:** aldehyde dehydrogenase, association study genetic, single nucleotide polymorphism (SNP), keratoconus (KC)

## Abstract

Background: ALDH3A1 protein is important in maintaining corneal physiology and protecting the eye from UV damage. However, none of the genome-wide association studies has indicated that the *ALDH3A1* locus is associated with keratoconus. In this study, we examined the potential role of *ALDH3A1* variants as risk factors for keratoconus incidence and severity in a large group of Polish keratoconus patients. Methods: In the first stage we analyzed the coding region sequence of the *ALDH3A1* in a subgroup of keratoconus. Then, we genotyped three selected *ALDH3A1* variants in a larger KC group of patients (*n* = 261) and healthy controls (*n* = 317). Results: We found that the rs1042183 minor allele A is a risk factor for keratoconus in the dominant model (OR = 2.06, 95%CI = 1.42–2.98, *p* = 0.00013). The rs2228100 variant genotypes appear to be associated with an earlier age of KC diagnosis in the Polish population (*p* = 0.055 for comparison of three genotypes and *p* = 0.022 for the dominant inheritance model). Conclusions: The rs1042183 variant in *ALDH3A1* is associated with keratoconus risk in the Polish population. The differences in the allele frequency between both populations could be partially responsible for the difference in the disease prevalence.

## 1. Introduction

Keratoconus (KC) is a major clinical eye disorder and the most common indication for corneal transplantation. It is a progressive, degenerative, mostly asymmetric disease. KC results in the weakening of the cornea and is caused by a complex collagen disorder distinguished by steepening of the cornea shape with irregular astigmatism or high myopia leading to major visual impairment. It is a multifactorial phenomenon of unclear aetiology with a substantial input from environmental factors. The prevalence of KC is usually estimated at 1.38 per 1000 [95% confidence interval (CI): 1.14–1.62 per 1000] [1] but recently it has been observed that its geographical distribution is not uniform. In Northern Europe, the Ural region, Japan and Northern USA, the prevalence is low, while India, China and the Middle East show relatively high prevalence [2]. A coincidence with hot and sunny conditions has been discussed. Chronic sun exposure and consequent oxidative damage induced by ultraviolet light could play a major role in the incidence of KC. On the other hand, other studies suggest variation in KC incidence among ethnic groups of the same country [2]. For instance, in Singapore, corneal steepness is much more frequent in the Indian population than in the Chinese or Malays [3], while in the English Midlands, Caucasians show 4.4 times lower KC incidence than Asians [4]. Such differences, and also those in the age of onset of the disease, suggest a non-negligible genetic component to the KC aetiology, which underscores its multigenic character.

The genetic basis of KC is probably complex, involving numerous genes and their variants. It has been suggested that between five and twenty-three per cent of KC patients have a family history of the disease, but no specific causal genes have been identified [5]. However, the ZNF469 (zinc finger protein 469) gene involved in the regulation of expression of several extracellular matrix components, as well as COL5A1 (collagen alpha-1(V) chain), have been suggested to contribute to KC pathology. Moreover, a contribution of mitochondrial aberrations is well documented, one of the examples being *IMMP2L* coding for a mitochondrial inner membrane protease, mutations in which lead to enhanced oxidative stress (OS) [6]. Genome-wide association studies (GWAS) have led to the identification of more than 60 genes/loci associated with KC, including those involved in protection against OS [7], but no gene could be called causative [8]. Additionally, the clinical phenotype of KC has been associated with some genetic syndromes (e.g., Down, Ehlers–Danlos, Noonan and Marfan syndromes) [5].

A wide variety of growth factors, chemokines, cytokines and their receptors are synthesized by corneal epithelial and stromal cells and are found in tears, playing a major role in corneal wound healing and inflammatory response pathways. Proteoglycans and glycoproteins are essential for normal corneal function, both at the air–epithelium interface and within the extracellular matrix. Lumican, keratocan and mimecan are the major keratan sulfate proteoglycans of the corneal stroma. Along with interfibrillar proteins, including collagen type VI and XII, they are critical for the maintenance of corneal transparency [9]. There is ample evidence that the ALDH3A1 (aldehyde dehydrogenase 3 family, member A1) protein is important in maintaining corneal physiology. It represents up to 50% of the corneal water-soluble proteins in mammals. The cornea serves as a physical and biochemical protective barrier and the first defence against environmental stress to internal ocular tissues. Corneal ALDH3A1 protects the eye from ultraviolet (UV) damage by metabolizing the toxic aldehydes produced by UV-induced lipid peroxidation such as 4-hydroxy-2-nonenal. Additional functions of ALDH3A1 include direct absorption of UV radiation, chaperone-like activity, scavenging of UV-generated reactive oxygen species (ROS) via the -SH group of cysteine residues and generation of NADPH, which is the reducing agent used by the glutathione peroxidase/glutathion peroxidase system [10]. Exposure to ROS can cause OS and damage cells by reacting with proteins, DNA, and membrane lipids. The healthy cornea can effectively eliminate the ROS and prevent OS before it damages the cells, but when the defence systems are overwhelmed by an excessive environmental assault and/or malfunctioning of the system, numerous OS-induced lesions may develop, including corneal pathologies [11,12,13], lense cataracts [14], and macular degeneration [15]. Earlier studies have shown that the *ALDH3A1* knock-out mice develop premature cataracts (lens opacification), convincingly demonstrating the importance of this protein in preventing ocular toxicity [16]. Several recent reports suggest that the accumulation of ROS and different reactive aldehydes formed following ROS-induced lipid peroxidation and subsequent action of the antioxidant defence systems may play a role in the pathogenesis of KC [12,17,18]. Despite the extensive data on the likely involvement of ALDH3A1 in KC [19], none of the GWAS carried out to date have indicated that the *ALDH3A1* locus is associated with KC [7]. However, two preliminary results from the Polish [20] and Korean populations [21] suggested an association of *ALDH3A1* variants with KC risk. The preliminary data from the Korean population were confirmed recently and the rs2228100 variant of *ALDH3A1* was found to be strongly associated with KC risk [22].

All of this indicates that *ALDH3A1* is a determinant of the keratoconus pathology. In this study, we investigated the potential role of *ALDH3A1* variants as risk factors for KC incidence and severity. In a group of KC patients, we sequenced the coding region of *ALDH3A1*, finding several SNPs. We then genotyped three of those variants in a larger group of KC patients (*n* = 261) and healthy controls (*n* = 317).

## 2. Results

### 2.1. Patient Characteristics

The mean age of KC patients (*n* = 261) was 40 ± 13 years (range 17–79), whereas that of controls (*n* = 317) was 23 ± 2 years (range 20–36) with a higher percentage of males: 72% in KC and 54% in the control group. The mean age in KC patients was 38 ± 12 years for males and 46 ± 13 years for women. The maximum corneal curvature was 51.9 ± 6.1D (range 39–73D), mean thinnest corneal thickness was 440 ± 60 µm (range, 270–540 µm), mean corneal astigmatism was 4.8 ± 2.5D (range, 0.9–15D), and mean posterior BFS (floating best fit sphere) elevation was 55.2 ± 5.1 µm (range, 46.1–70.7 µm).

### 2.2. Sequencing and SNP Calling

Sequencing of the *ALDH3A1* exons with flanking intronic sequences and the promoter region in the pilot group (*n* = 28 KC patients) revealed eleven known variants. All variants but one (rs4646791- single nucleotide insertion in intron 8) are single nucleotide polymorphism changes. Three variants were intronic, one was located in the 3’UTR, and six (three synonymous and three missense) were located in the protein-coding sequence (Appendix A Table A1).

By comparing the frequency of the variants in the 28 KC patients with their frequencies in non-Finnish Europeans according to Gnomad [23] and our own sequencing data for diverse groups representing the Polish population, we selected three SNPs (rs141568499, rs2228100, rs1042183) apparently over-represented in the KC patients for further examination in the whole KC cohort (*n* = 261) vs. control group (*n* = 317). The distribution of the three SNPs in the KC and control groups is shown in Table 1. The frequencies of the genotypes for all the variants did not deviate from Hardy–Weinberg equilibrium in the control group (*p* > 0.05). One SNP (rs1042183) deviated from the Hardy–Weinberg equilibrium in the KC group (*p* = 0.00003) with an excess of heterozygotes (62.7%). The presence of at least one minor allele A of rs1042183 (dominant model) was significantly associated with a higher risk of KC (OR = 2.06, 95%CI = 1.42–2.98, *p* = 0.00013), while in the additive model, the association of rs1042183 A alleles did not reach statistical significance (OR = 1.25 per each A allele, 95%CI = 0.99–1.57, *p* = 0.066). The two other SNPs (rs141568499 and rs2228100) showed no statistically significant association with the KC (KC patients vs. control group).

Statistical analysis comparing three genotype groups with the Kruskal–Wallis test did not reveal any statistical significance between alleles distribution of the analyzed SNPs and age of diagnosis and phenotype topographic features in the KC patients group. However, when comparing the two genotype groups according to the dominant inheritance model, a significant association was observed between rs2228100 and age of disease diagnosis and thinnest corneal thickness. Compared with wild-type GG homozygotes, carriers of the minor C allele were diagnosed earlier (age of diagnosis: 38.3 ± 11.6 vs. 41.9 ± 13.0 years, *p* = 0.022) and had greater corneal thickness (446 ± 54 µm vs. 427 ± 62 µm, *p* = 0.042). In the dominant model, we also observed a borderline significant association between rs141568499 and maximum corneal curvature, which was slightly higher in carriers of the minor C allele than in AA homozygotes (54.1 ± 6.0D vs. 51.7 ± 6.0D, *p* = 0.071).

## 3. Discussion

ALDH3A1 is an important detoxifying enzyme in corneal epithelial cells comprising up to 40% of the soluble protein content of mammals and has been proposed to be involved in keratoconus aetiology [19]. The *ALDH3A11* gene is among those with the highest expression level in corneal epithelium (the top 2% ranked by the *ALDH3A1* gene are among those with the highest expression level in corneal epithelium (top 2% ranked by reads per kilobase of transcript, RPKT)) [24]. While no genome-wide association studies (GWAS) have indicated an association of the *ALDH3A1* with KC [7], it has been noted in intraocular pressure [25] and refractive astigmatism studies [26] (https://www.ebi.ac.uk/gwas/genes/ALDH3A1) (accessed on 6 June 2021) [7]. Astigmatism can be one of the first signs of KC. Serdarogullari et al. [27] showed that as many as 14.1% of patients in a general ophthalmology outpatient clinic with astigmatism of 2D or greater had some degree of KC. ALDH1A1 constitutes 3 and 2% of soluble proteins in the cornea and lens epithelium in the human, respectively, and, together with ALDH3A1, protects inner ocular tissues from ultraviolet radiation and reactive oxygen-induced damage [28].

This study is an extension of our results presented at the Association for Research in Vision and Ophthalmology (ARVO) annual meeting 2016 [20] for reporting the first time an association has been found of *ALDH3A1* variants with KC in Poles. In addition, a recent study of the Korean population reported such an association (rs2228100 strongly associated with KC risk) [22].

We identified the minor A allele of rs1042183 as a risk factor for KC (in a dominant model). This variant is located in the 3′ untranslated region (3′ UTR) of the canonical ALDH3A1 transcript (ENST00000457500.2) with nucleotide G as the ancestral and A as the minor allele. The minor allele is actually common in European (minor allele frequency, MAF = 0.42, minor allele frequency) and African (MAF = 0.11) populations and is absent in South Asia populations according to gnomaAD (date: 2 August 2021). In our control group, MAF was 0.41, and in the group of patients with KC, it was 0.47. In the Polish population, the odds ratio for the occurrence of KC in the carrier of at least one A allele compared to GG homozygotes is 2.06.

The rs2228100 variant genotypes appeared to be associated with an earlier age of KC diagnosis in the Polish population (*p* = 0.055 for comparison of three genotypes and *p* = 0.022 for the dominant inheritance model). This SNP is a missense mutation (P329A, C is the minor allele) located in exon 8. In our study, both patient and control group MAF was ca. 0.27, similar to the gnomAD data for the European population (MAF = 0.25). Interestingly, this MAF is higher in South Asian and African populations (0.39 and 0.47 respectively). This SNP was also strongly associated with the risk of KC in the Korean population (C/C genotype OR = 2.1; C allele, OR = 1.6) [21].

Unlike the Korean study, we did not observe any association of rs2228100 with a predisposition for KC. The rs2228100 allele distribution C vs. G had an OR = 1.06 (95%CI = 0.81–1.37) in the present study, an OR = 1.81 (95%CI = 1.308–2.490) in the Korean study, and, in the dominant inheritance model (CC + CG vs. GG), OR = 1.01 (95%CI = 0.73–1.41) (Polish) compared to OR = 4.35 (95%CI = 1.99–9.35) (Korean). Since the confidence intervals for the OR in the dominant model do not overlap between the Korean and Polish populations, the genotype–phenotype association is apparently strongly dependent on ethnicity. However, in the Polish study, we observed that allele C in the dominant inheritance model, associated with earlier age of diagnosis and higher thinnest value in the Polish population, fits into the influence of rs2228100 with KC. This observation in distinct populations with a significant difference in distributions of alleles may suggest a direct, functional effect at the molecular level associated with KC.

According to gnomAD, rs141568499 is an infrequent variant in the European (Finnish) population or a rare variant in the other populations (Finnish MAF = 0.016, non-Finnish European MAF = 0.008 and other populations MAF < 0.005). It is a missense p.E270A mutation, benign according to Polyphen but deleterious according to SIFT (Sorting Intolerant From Tolerant) software. We observed a trend to higher values of the maximum corneal curvature associated with the minor allele. Visual limitations are strongly correlated with the maximum corneal curvature. Sharp visual acuity requires light to be focused onto the photoreceptor layer of the retina by a combined role of the cornea and the crystalline lens. The cornea is the major refracting element, and its curvature form and asymmetry may impair vision by causing refractive errors, manifesting as myopia or astigmatism. Elevated corneal steepness is a hallmark feature of KC. Thus, a protection potential of the AA genotype in KC cannot be excluded [29].

The MAF values of all the analyzed variants of *ALDH3A1* differ worldwide, and, in addition, the KC prevalence is variable in different parts of the world, likely due to diverse environmental and ethnic factors [30]. A large-cohort study reported substantial ethnic differences in KC prevalence in the USA; Afro-Americans had 57% higher odds than Latino persons (43%), whereas Asians had 39% reduced odds of being diagnosed with KC compared with Whites [31]. One is tempted to speculate that the common variants of *ALDH3A1* could explain, at least in part, the ethnically varied distribution of KC prevalence. Thus, the MAF for rs1042183 (A) is 0.42 for Europeans and 0.11 for African/African-Americans (gnomaAD), and, for rs2228100 (shown to be a strong risk factor for KC), it varies between 0.47 in African/African-Americans and 0.25 in Non-Finish Europeans (gnomAD).

A limitation of the present study is that the control group was not matched to the study group in regards to age and sex, so it can be viewed as a population control only. However, this should not affect the genotype-phenotype associations in the case of a rare disease (with a very low probability of a control subject developing KC in the future) and in the absence of any indication suggesting variation in the distribution of the *ALDH3A1* gene dependent on age or sex. The likelihood that a particular control individual will develop KC in the future is statistically negligible (with the prevalence of KC estimated at 1.38 per 1000 in the general population, the probability that one of 317 controls will ever develop KC is less than 1/3). If, on the other hand, for a control group aged >60–70 years, the frequency of variants could be influenced by mortality. Regarding sex-related stratification, the results from time to time indicate that the ALDH3A1 variants are not sex-related. We believe that the control group reflects the frequency of the genetic variant in the entire population.

## 4. Materials and Methods

### 4.1. Participants and Clinical Data

A total of 261 KC patients and 317 healthy controls were examined. The subjects were recruited in 2012–2013 after receiving standard, regular ophthalmological monitoring. KC diagnosis was based on clinical examination (refractive errors, stromal thinning, Vogt’s striae, Munson’s sigh, Fleischer’s ring, etc.) and specific corneal topographic features based on Rabinowitz’s criteria, i.e., posterior corneal elevation within the central 3 mm ≥ +20 μm, inferior-superior dioptric asymmetry (I-S value) > 1.4 diopters (D), the steepest keratometry > 47.2D. All measurements were performed with a Placido-based topography (Orbscan II; Bausch & Lomb, Rochester, NY, USA). In case of uncertain topographic results, the examination was repeated 15 min later after the administration of artificial teardrops containing hyaluronic acid. To reduce the impact of diurnal variations, all imaging tests were performed between 10:00 and 16:00 and at least 4 h after the patient’s waking time. A patient was considered KC-positive when their KC percentage index (keratometry, I-S, skew percentage, astigmatism, %KISA), specific and sensitive index for determining the KC, was higher than 100% in at least one of the eyes. Clinically, the patient group was highly heterogeneous, varying from subclinical cases to severe ones resulting in a corneal transplant. Patients with diagnosed metabolic disorders were excluded.

### 4.2. Sequencing and SNP Calling

DNA was extracted from peripheral blood lymphocytes and isolated using an Extractme genomic DNA kit according to the manufacturer’s protocol (Blirt, Gdańsk, Poland).

The promoter region and exons 1–10 with flanking intron sequences of *ALDH3A1* gene (NG_012251.1) from 28 KC patients were sequenced using the Sanger method (Abi 3130, Thermo Fisher, Waltham, MA, USA). Primers for amplification and sequencing were designed using Primer 3 Input ver.0.4.0 [32,33] (http://bioinfo.ut.ee/primer3-0.4.0/) (accessed on 6 June 2021) (Appendix A Table A2).

Then three selected SNPs (rs141568499, rs2228100, rs1042183) were genotyped in KC patients and healthy controls using TaqMan^®^ SNP Genotyping Assays (Thermo Fisher), KAPA PROBE FAST Universal qPCR Kit (Kapa Biosystems, Bath, UK) and StepOnePlus™ Real-Time PCR System (Thermo Fisher). The results were analyzed using StepOne Software v2.2.2.

### 4.3. Statistical Analyses

A chi-squared test was used to compare genotype and allele frequencies between the study and the control group. The Kruskal–Wallis or Mann–Whitney test was used to compare quantitative parameters between genotype subgroups within the study group. Concordance of genotype distribution with Hardy–Weinberg equilibrium (HWE) was verified in each group with an exact test. *p* < 0.05 was considered statistically significant.

## 5. Conclusions

We showed that the rs1042183 variant in the *ALDH3A1* gene is associated with a predisposition to keratoconus in the Polish population. More precisely, allele A of rs1042183 is strongly associated with the risk of keratoconus in a dominant model. Allele frequency of *ALDH3A1* variants associated with KC varies between distinct populations, which can be partially responsible for the difference in KC prevalence worldwide.

## Figures and Tables

**Table 1:** Distribution of genotypes and alleles *ALDH3A1* gene polymorphisms in the KC patients and the controls.

rs141568499											
Group		Genotypes			Alleles		Comparisons *	OR **	95%CI		*p* ***
		AA	AC	CC	A	C					
Controls	*n*	290	25	2	605	29	CC + AC vs. AA	1.04235	0.582431	1.865449	0.888909
	*%*	91.48	7.89	0.63	95.42	4.57	CC vs. AC + AA	0.608108	0.054832	6.744155	0.682313
KC patients	*n*	237	22	1	496	24	C vs. A	1.009455	0.580235	1.756183	0.973428
	*%*	91.15	8.46	0.38	95.38	4.61					
**rs2228100**											
Group		Genotypes			Alleles		Comparisons *	OR **	95%CI		*p* ***
		GG	GC	CC	G	C					
Controls	*n*	173	123	21	469	165	CC + GC vs. GG	1.013938	0.729489	1.409302	0.93433
	*%*	54.57	38.8	6.62	73.97	26.03	CC vs. GC + GG	1.302921	0.699627	2.426442	0.40317
KC patients	*n*	141	97	22	379	141	C vs. G	1.057472	0.813413	1.374759	0.676369
	*%*	54.23	37.31	8.46	72.88	27.12					
**rs1042183**											
Group		Genotypes			Alleles		Comparisons *	OR **	95%CI		*p* ***
		GG	GA	AA	G	A					
Controls	*n*	116	140	61	372	262	AA + GA vs. GG	2.055337	1.416549	2.982186	0.00013
	*%*	36.59	44.16	19.24	58.68	41.32	AA vs. GA + GG	0.76304	0.492615	1.181917	0.224944
KC patients	*n*	57	163	40	277	243	A vs. G	1.24557	0.985888	1.573652	0.065503
	*%*	21.92	62.69	15.38	53.27	46.73					

* Comparison of genotype or allele frequencies between the KC patient and control groups. ** OR for the genotype or allele frequencies compared between KC patient and control groups. *** Chi-square test.

## Data Availability

Data available on request.

## Appendix A

**Table A1:** *ALDH3A1* variants identified in the pilot group of KC patients (*n* = 28) (data from: Variant Effect Predictor, ensemble.org; Variant Effect Predictor—Homo_sapiens—Ensembl genome browser 104).

#Uploaded Variation	Location	Consequence	EXON	INTRON	HGVSc	HGVSp	cDNA Position	CDS Position	Protein Position	Amino Acids	Codons	SIFT	PolyPhen	AF	EUR AF	gnomAD AF	gnomAD NFE AF
rs56234923	17:19744906-19744906	intron variant	-	1	c.162 + 62A > G	-	-	-	-	-	-	-	-	-	-	-	-
rs59102760	17:19743437-19743437	synonymous variant	2	-	c.189G > A	p.Glu63=	519	189	63	E	gaG/gaA	-	-	0.0116	0.0268	0.01945	0.02135
rs887241	17:19742625-19742625	missense variant	3	-	c.400T > G	p.Ser134Ala	730	400	134	S/A	Tca/Gca	Tolerated (0.88)	Benign (0)	-	0.6441	0.7057	0.6638
rs2072328	17:19742310-19742310	intron variant	-	3	c.481−98C > T	-	-	-	-	-	-	-	-	0.0429	0.008	-	-
rs2072330	17:19741159-19741159	synonymous variant	5	-	c.741T > A	p.Pro247=	1071	741	247	P	ccT/ccA	-	-	0.2907	0.3907	0.3577	0.4023
rs3826507	17:19740497-19740497	intron variant	-	5	c.808−20C > T	-	-	-	-	-	-	-	-	0.2941	0.3917	0.359	0.3993
rs141568499	17:19740476-19740476	missense variant, splice region variant	6	-	c.809A > C	p.Glu270Ala	1139	809	270	E/A	gAg/gCg	Deleterious (0.03)	Benign (0.076)	0.0004	0.001	0.004717	0.006718
rs2228100	17:19739639-19739639	missense variant	7	-	c.985C > G	p.Pro329Ala	1315	985	329	P/A	Ccg/Gcg	Tolerated (0.17)	Benign (0)	0.3800	0.2396	0.3115	0.249
rs4646791	17:19738956-19738956	intron variant	-	8	c.1216 + 40_1216 + 41insC	-	-	-	-	-	-	-	-	-	0.4006	0.3846	0.4093
rs57555435	17:19738431-19738431	synonymous variant	9	-	c.1239C > T	p.Tyr413=	1569	1239	413	Y	taC/taT	-	-	0.0369	0.0089	0.02118	0.007902
rs1042183	17:19738008-19738008	3 prime UTR variant	10	-	c.*213G > A	-	1905	-	-	-	-	-	-	0.2965	0.3917	-	-

**Table A2:** Primers used for PCR and Sanger sequencing.

Exon	Primer F	Primer R
promoter region	AAACAGCCCGGGACCTAAT	AGGAATGCAGGGAAGAGGAT
promoter region	TTTCGTGTATTGGTAGCAGGA	GTCCTGCCCGAGCTGAAG
1	CCAGGGAAGTCCCTTCCTAT	CCCAAGTCCTTCCTGAACCT
2	ATCACCCTGGCAGCTGAG	ATCCTGGCTTGGCTCCAC
3 + 4	AGAGCCTCTGGTGAAGTACCC	CCCCTACCCCCATGTAAGTT
5	GTCTGCAAAACGAGATGCAA	CCAAGGGGTCAACACTCACT
6	CTGTGTCTGTGCAGCCTTTG	CTGTGCTCTTCAGGGGTCAC
7	AGGTGCTTGCACTTTCCATC	CCTGAATTTGCTGGGTGAGT
8	ACCTGCTCCTGTCCTTGTG	CCTGAATTTGCTGGGTGAGT
9 + 10, 3′UTR	ACACCCAACAAGGTGGAGAG	ATGAGAGGCCTTTGCTGGTA
